# Lysophospholipid-Related Diseases and PPARγ Signaling Pathway

**DOI:** 10.3390/ijms18122730

**Published:** 2017-12-16

**Authors:** Tamotsu Tsukahara, Yoshikazu Matsuda, Hisao Haniu

**Affiliations:** 1 Department of Pharmacology and Therapeutic Innovation, Nagasaki University Graduate School of Biomedical Sciences, 1-14 Bunkyo-machi, Nagasaki 852-8521, Japan; 2 Clinical Pharmacology Educational Center, Nihon Pharmaceutical University, Ina-machi, Saitama 362-0806, Japan; yomatsuda@nichiyaku.ac.jp; 3 Institute for Biomedical Sciences, Interdisciplinary Cluster for Cutting Edge Research, Shinshu University, 3-1-1 Asahi, Matsumoto, Nagano 390-8621, Japan; hhaniu@shinshu-u.ac.jp

**Keywords:** lysophospholipids, PPARγ, vascular diseases, dementia, spinal cord injury

## Abstract

The nuclear receptor superfamily includes ligand-inducible transcription factors that play diverse roles in cell metabolism and are associated with pathologies such as cardiovascular diseases. Lysophosphatidic acid (LPA) belongs to a family of lipid mediators. LPA and its naturally occurring analogues interact with G protein-coupled receptors on the cell surface and an intracellular nuclear hormone receptor. In addition, several enzymes that utilize LPA as a substrate or generate it as a product are under its regulatory control. Recent studies have demonstrated that the endogenously produced peroxisome proliferator-activated receptor gamma (PPARγ) antagonist cyclic phosphatidic acid (cPA), which is structurally similar to LPA, inhibits cancer cell invasion and metastasis in vitro and in vivo. We recently observed that cPA negatively regulates PPARγ function by stabilizing the binding of the co-repressor protein, a silencing mediator of retinoic acid, and the thyroid hormone receptor. We also showed that cPA prevents neointima formation, adipocyte differentiation, lipid accumulation, and upregulation of PPARγ target gene transcription. The present review discusses the arbitrary aspects of the physiological and pathophysiological actions of lysophospholipids in vascular and nervous system biology.

## 1. PPARγ and Lysophospholipids

Phospholipids are hydrolyzed by phospholipase A_2_ (PLA2) to produce lysophospholipids and free fatty acids. One of the most attractive targets of PLA2 is lysophosphatidic acid (LPA), a naturally occurring phospholipid that functions as a bioactive lipid mediator and a second messenger [1]. It consists of a glycerol backbone with a hydroxyl group, a phosphate group, and a long-chain saturated or unsaturated fatty acid. LPA has been detected in biological fluids, and it performs a wide range of biological functions in cell proliferation, migration, and survival [2,3]. LPA is produced by platelet activation after activation of multiple biochemical pathways [4,5]. The plasma contains nanomolar quantities of LPA, whereas LPA concentration can reach physiological levels in the serum during blood clotting [6,7]. LPA has attracted considerable interest because of its multiple roles in physiological and pathological conditions. Recent studies suggest that LPA receptor (LPAR) antagonists abolish platelet aggregation elicited by mildly oxidized low-density lipoprotein (LDL) (mox-LDL), indicating that an LPA-like lipid plays an essential role in the thrombogenic effects of mox-LDL [8]. LDL oxidation generates peroxisome proliferator-activated receptor (PPAR)γ agonists [9], including alkyl glycerophosphate (AGP) [10]. AGP is also formed enzymatically from alkyl dihydroxyacetone phosphate [11]. AGP concentration in the brain is 0.44 nmol/g, which is 15% that of acyl-LPA [12]. Here, we provide evidence that AGP is a PPARγ ligand, with potency similar to that of the thiazolidinedione rosiglitazone, but with only 40% efficacy. Computational and mutational analysis of the AGP-PPARγ complex indicates differential interaction with key residues in the ligand binding and activation domains that explains the partial activation elicited by AGP. Several reports have identified putative intracellular agonists of PPARγ. For example, selected forms of LPA, which accumulate as oxidatively modified LDL, also activate PPARγ [13]. LPA exerts growth-like effects in almost every mammalian cell type. Although LPA is the known ligand for G-coupled cell surface LPARs, some of the effects of LPA are also mediated by PPARγ activation [8]. PPARγ plays key roles in regulating lipid and glucose homeostasis, cell proliferation, apoptosis, and inflammation. In contrast, cPA, which is structurally similar to LPA, is generated by phospholipase D2 (PLD2) and negatively regulate PPARγ functions [14]. cPA shows several unique functions compared to LPA [15]. Unlike LPA, cPA inhibits cell proliferation [16]. Reports show that cPA attenuates neointima formation, which is an early step in the development of atherosclerotic plaques [17]. cPA is a second messenger and a physiological inhibitor of PPARγ, revealing that PPARγ is regulated by both agonists and antagonists.

## 2. Lysophospholipid and Vascular Pathologies

LPA has been identified as a platelet-activating lipid of mox-LDL in human atherosclerotic lesions [8]. Relatively few intracellular binding partners for LPA are known. Previous studies have identified some candidate proteins, including C-terminal-binding protein/brefeldin A-dependent ADP ribosylated substrate [18], liver fatty-acid-binding protein [19], and gelsolin [20]. Recently, we reported that the isolation and purification of heart-type fatty-acid-binding protein (FABP3) from human coronary artery endothelial cells (HCAECs) were coupled to their identification by proteomics techniques [21]. FABP3, a small cytoplasmic protein with a molecular mass of about 15 kDa, transports fatty acids and other lipophilic substances from the cytoplasm to the nucleus, where these lipids are released to a group of nuclear receptors such as PPARs [21]. FABP3 did not bind LPC or activate PPARγ in HCAEC, showing that FABP3 distributes from the cytosol to the nucleus in response to LPA-mediated PPARγ activation. Recent reports showed that AGP plays an important role in the vascular system [22]. Our group reported that AGP activates PPARγ-mediated transcription more than LPA [10]. Activation of biochemical pathways linked to platelet activation induces AGP production in the serum [8]. Binding studies using the PPARγ ligand-binding domain (LBD) showed that the binding affinity of AGP to PPARγ was similar to that of the synthetic agonist, rosiglitazone [10]. AGP has been detected in several biological fluids and tissues, including the human brain, ascitic fluid, and saliva [23,24,25]. Recently, we identified that AGP and rosiglitazone induce neointima formation when applied topically within the carotid artery [14]. Neointimal lesions are characterized by the accumulation of cells within the arterial wall and are a prelude to atherosclerotic disease [8]. Recent reports showed that the knockdown of the gene encoding 1-acyl-sn-glycerol-3-phosphate acyltransferase β (AGPAT2) increased cPA levels [26]. AGPAT2 is located the endoplasmic reticulum membrane and converts LPA to phosphatidic acid (PA). Mutations in *AGPAT2* have been associated with congenital generalized lipodystrophy (CGL) [26,27]. Lipodystrophies, including CGL, are heterogeneous acquired or inherited disorders characterized by the selective loss of adipose tissue and development of severe insulin resistance. Histone deacetylases (HDACs), which have been shown to activate PPARγ and enhance the expression of its target genes, regulate chromatin structure and gene transcription via interactions with nuclear receptor corepressors, such as SMRT and nuclear receptor corepressor (NCoR) [28]. HDAC3 inhibits PPARγ and nuclear transcription factor-κB (NF-κB) [29], and HDAC3 inhibition restores PPARγ function in obesity [30]. Additionally, HDAC2-containing complexes are involved in the regulation of nuclear receptor-dependent gene transcription [31]. A previous study demonstrated that topical application of AGP onto uninjured carotid arteries of rats induces arterial wall remodeling in a PPARγ-dependent manner [14]. Our current study also identified increased AGP levels in the carotid artery of *apoE*^−/−^ mice [32]. These results suggest that AGP in the circulatory system may be a risk factor for development of diabetes-mediated atherosclerosis.

## 3. Lysophospholipids and Vascular Dementia

The brain is a lipid-rich organ, the structure and function of which are influenced by diet and nutrients [33]. Bioactive lipids within the brain are shown to be pivotal for central nervous system homeostasis by modulating neurotransmission, synaptic plasticity, enzyme function, ion channel activities, gene expression, and inflammation [34]. Lysophospholipids are also involved in a variety of important processes, including vascular dementia. Vascular dementia is a progressive disease caused by reduced blood flow to the brain, and it affects cognitive abilities especially executive function [35,36]. Vascular dementia is poorly understood, and the dearth of suitable animal models limits the understanding of the molecular basis of the disease and development of suitable therapies [36]. On the basis of their chemical structures, different bioactive lysophospholipids can be assigned either to the group of lysophospholipids, LPA, and lysophosphatidylcholine (LPC), or the group of lysosphingolipids, lysosphingomyelin (SPC), and sphingosine 1-phosphate (S1P). LPA is present in the embryonic brain, neural tube, spinal cord, and cerebrospinal fluid at nanomolar to micromolar concentrations and plays several significant roles in the nervous system during development and injury [34]. In the adult brain, LPA receptors are differentially expressed in various neural cell types; for example, the LPA_1_ receptor affects cerebral cortical neuron growth, growth cone and process retraction, survival, migration, adhesion, and proliferation [37]. Our recent study suggested that LPA treatment profoundly induced the expression of Kruppel-like factor 9 (KLF9) in human induced pluripotent stem cell-derived neurons [38]. Furthermore, we observed that the effects of LPA on neurite outgrowth and proliferation were also mediated through the PPARγ pathway [38]. Studies show that KLF9, a member of the KLF family of evolutionarily conserved zinc finger transcription factors [39], has been implicated in mediating a diverse range of biological processes including neural stem cell maintenance [40]. *KLF9* expression is induced by neuronal activity as dentate granule neurons functionally integrate in the developing and adult dentate gyrus (DG). During brain development, dentate granule neurons lacking KLF9 show delayed maturation as reflected by the altered expression of early-phase markers and dendritic spine formation [41,42]. Adult *KLF9*-null mice exhibit normal stem cell proliferation and cell fate specification in the DG but show impaired differentiation of adult-born neurons and decreased neurogenesis-dependent synaptic plasticity [41]. Although further investigations will be needed to ascertain the underlying mechanism, these reports highlight that the KLF9-LPC axis is essential for neuronal development. The presence of PPARs has been extensively studied in nervous tissue [43]; PPARs are present in astrocytes, oligodendrocytes, microglia, and neural stem cells (NSCs) [44,45,46,47], where it inhibits proinflammatory gene and protein expression. For example, PPARγ inhibits proinflammatory transcription factors, nuclear factor-κB (NF-κB) [48], and activator protein 1 (AP-1) [49]. Owing to the anti-inflammatory and potentially neuroprotective effects of PPARγ, PPARγ agonists are increasingly being used for the treatment of neurodegenerative diseases [50]. Since PPARγ does not colocalize significantly within microglia, several studies indicated a reduction in microglial activity after PPARγ agonist administration [51]. A recent study suggested that LPC, a precursor of LPA, exerts direct biological effects, especially on vascular dementia [52,53]. Plasma LPC is produced by lecithin-cholesterol acyltransferase, hepatic secretion, or by the action of phospholipase A2 (PLA2) [54]. PLA2 are enzymes that catalyze the cleavage of fatty acids from the sn-2 position of phospholipids, producing free fatty acids and LPC. However, abundant evidence exists regarding the capacity of free LPC to increase cytosolic Ca^2+^ and activate inflammatory signaling pathways [55]. In a study of the plasma metabolic profile of Alzheimer’s disease (AD), a decrease in LPC 16:0 and 18:2 was reported [56]. Furthermore, previous studies have suggested that oxidative stress is related to AD [57]. These stimulations can activate PC metabolism and downregulate LPC [58]. Therefore, it is important to further evaluate the significance of targeting these bioactive lipids.

## 4. Lysophospholipids and Spinal Cord Injury (SCI)

A recent estimate shows that the annual incidence of spinal cord injury (SCI) is approximately 54 cases per one million people in the United States, or about 17,500 new SCI cases per year [59]. SCI results in serious damage at the site of injury in the initial stages of neurotrauma, and is complicated by the inflammatory response, which prevents neuronal regeneration and recovery by the central nervous system (CNS) [60]. In addition, a considerable extent of the post-traumatic degeneration of the spinal cord is due to a multifactorial secondary injury [61]. Currently, therapeutic research is focused on two main areas—neuroprotection and neuroregeneration. Several therapeutic strategies have been developed to potentially intervene in these progressive neurodegenerative events and minimize secondary damage to the spinal cord. A variety of promising drugs have been tested in animal models, but few can be applied on human patients with SCI. Neuroprotective drugs target secondary injury effects, including inflammation, oxidative stress-mediated damage, glutamate excitotoxicity, and programmed cell death. Several potentially neuroprotective agents that target the above pathways are under investigation in human clinical trials [62]. Reports show that blocking of LPA signaling is a useful and novel therapeutic strategy for SCI [63]. In the murine SCI model, the use of a specific anti-LPA monoclonal antibody indicated that LPA produced endogenously after neurotrauma inhibits SCI regeneration [63]. In the normal spinal cord, six different LPA receptors (LPA_1_-LPA_6_) were expressed constitutively, and LPA_1_ was the most highly expressed [64]. LPA leads to demyelination via activation of microglia LPA_1_. Moreover, we demonstrate that selective blockade of LPA_1_ after SCI reduces functional deficits and demyelination, altogether revealing important contributions of LPA–LPA_1_ signaling in secondary damage after SCI [64]. In addition, FTY720, an orally available sphingosine-1-phosphate (S1P) receptor modulator known clinically as fingolimod [65], protects an animal model of ischemia-reperfusion after cerebral ischemia and improves functional outcomes in a rat model of SCI. FTY720 is a first-in-class S1P receptor modulator that was highly effective in phase II clinical trials for multiple sclerosis. S1P is a bioactive lysophospholipid mediator that produces a variety of cellular responses, including proliferation, survival, and motility via association of the receptor with G protein-coupled receptor (GPCR) [66]. The efficacy of FTY720 in SCI is possibly because of its role in immune modulation. These studies suggest that lysophospholipids are key modulators of nervous system disorders, including SCI. Furthermore, PPARγ can potentially minimize or prevent dysfunction after SCI [67]. Increased intracellular calcium levels, mitochondrial dysfunction, arachidonic acid breakdown, and activation of nitric oxide synthase (NOS) immediately after SCI results in the formation of reactive oxygen (ROS) and nitrogen species (RNS) [67]. Treatment with the PPARγ agonist pioglitazone increased the number of motor neurons after SCI, which might partially reduce post-SCI oxidative damage [67]. However, none of the agents tested until now have demonstrated strong clinical beneficial outcomes in patients with SCI. Thus, the search for pharmacological drugs capable of improving neurological function is still on. Strategies targeted at modulating lysophospholipid levels in the injured CNS may lead to new therapeutic approaches toward repairing various CNS disorders.

## 5. Conclusions

**Figure 1:**
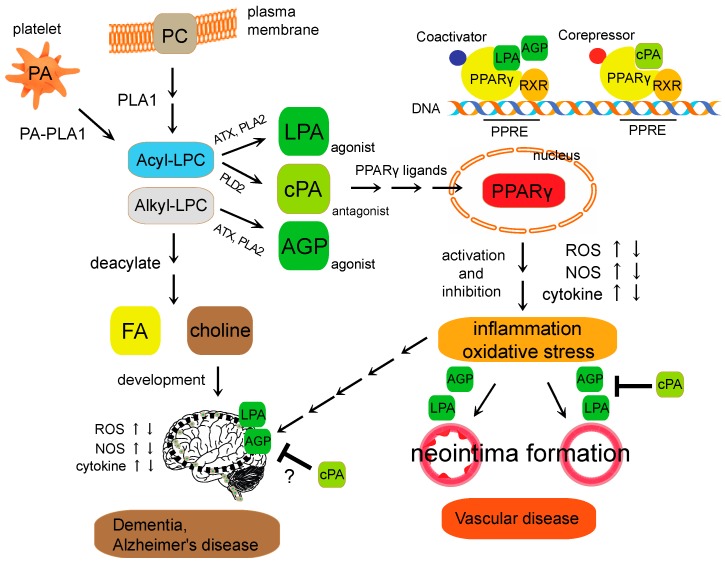
Schematic diagram of lysophospholipid-mediated PPARγ signaling. Lysophosphatidylcholine (LPC) is a bioactive phospholipid generated primarily by the action of phospholipase A2 (PLA2) enzymes on the plasma membrane. After cellular uptake, free LPC is reacylated yielding PC or deacylated yielding FA and choline. LPA and AGP are generated intracellularly in a stimulus-coupled manner by the ATX or PLA2 enzyme. cPA is generated intracellularly in a stimulus-coupled manner by the PLD2 enzyme. LPA and AGP induced neointima formation through the activation of PPARγ, whereas cPA inhibited PPARγ-mediated arterial wall remodeling in a noninjury infusion model. However, the physiological context of cPA in PPARγ signaling in brain is still unclear. Imbalance of the PPARγ agonist-antagonist equilibrium is involved in changes in cellular functions, including ROS generation, NOS and cytokine expression. These endogenous lysophospholipids regulate PPARγ function required for vascular wall pathologies, and metabolic-related diseases. PPRE (PPAR response element); RXR (retinoid X receptor); ATX (autotaxin).

In this review, we have focused on recent developments that elucidate the role of lysophospholipids in vascular and nervous system biology. Our proposed mechanism of action for lysophospholipid-related diseases is summarized in Figure 1. Lysophospholipids act as mediators via the activation of cell surface GPCRs, and as intracellular second messengers through PPARγ activation and inhibition in diseases such as atherosclerosis, dementia, and spinal cord injury. However, the physiological role of lysophospholipids in PPARγ signaling is still unclear; further understanding would promote the synthesis of novel medicines that modulate lysophospholipid-mediated PPARγ regulation.

## Abbreviations

PPAR	Peroxisome proliferator-activated receptor
LPA	Lysophosphatidic acid
AGP	1-*O*-octadecenyl-2-hydroxy-sn-glycero-3-phosphate
LPC	Lysophosphatidylcholine
cPA	Cyclic phosphatidic acid
S1P	Sphingosine 1-phosphate
SPC	Lysosphingomyelin
KLF9	Kruppel-like factor 9
mox-LDL	Mildly oxidized low-density lipoprotein
NF-κB	Nuclear factor-κB
AP-1	Activator protein 1
PLA2	Phospholipase A2
AD	Alzheimer’s disease
SCI	Spinal cord injury
ROS	Reactive oxygen species
RNS	Reactive nitrogen species
NOS	Nitric oxide synthase
CNS	Central nervous system
